# Supplementary figures and images for: Global change differentially modulates Caribbean coral physiology
Source: PLoS One. 2022 Sep 2;17(9):e0273897. doi: 10.1371/journal.pone.0273897 (PMC9439252; doi:10.1371/journal.pone.0273897)

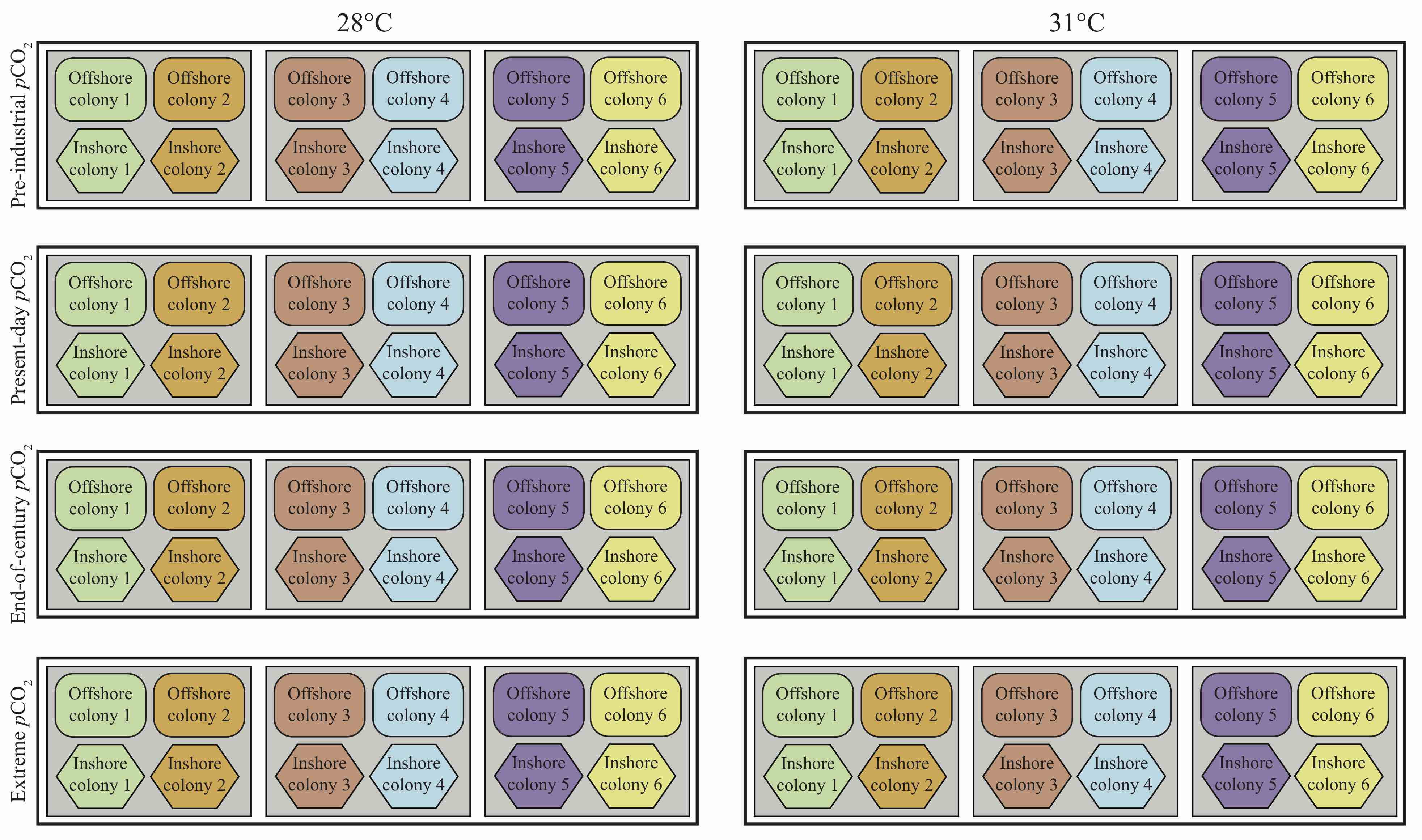

Supplement: S2 Fig — Diagram showing allocation of coral fragments for a single species throughout the experiment. Colour represents a different colony and shape represents reef environment. Four colonies (two from each reef environment) are reared within each tank (grey box), with three tanks comprising a treatment (white box). This is repeated for each pCO2 treatment at both temperatures. This same experimental design was used for all species. This figure is taken from Bove et al. 2019. (TIFF) [file pone.0273897.s002.tiff]

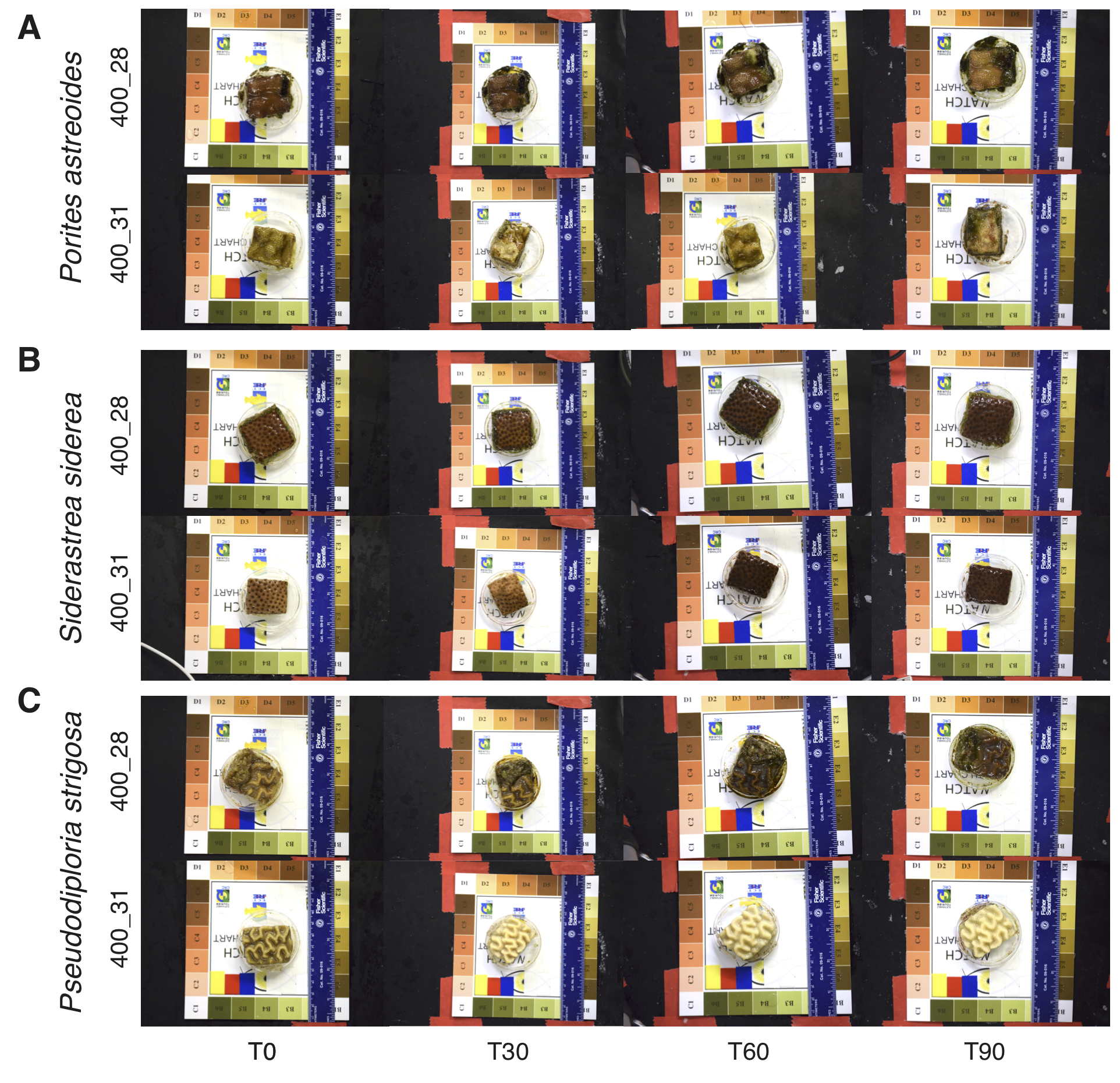

Supplement: S7 Fig — Coral colour changes over the experimental period. Representative images of fragments of (A) P. astreoides, (B) S. siderea, and (C) P. strigosa from the same colonies demonstrating change in coral colour over time in either control (420 μatm; 28°C) or warming (420 μatm; 31°C) treatments from the start of the experiment (T0) to the end (T90). (TIFF) [file pone.0273897.s007.tiff]
