# Supplementary material for: Global change differentially modulates Caribbean coral physiology
Source: PLoS One. 2022 Sep 2;17(9):e0273897. doi: 10.1371/journal.pone.0273897 (PMC9439252; doi:10.1371/journal.pone.0273897)
Supplement: S1 Text — (PDF) [file pone.0273897.s011.pdf]

Supplemental Tables for manuscript: *Global change differentially modulates Caribbean coral physiology*

Colleen B. Bove[1,2*], Sarah W Davies[1], Justin B Ries[3], James Umbanhowar[2,4], Bailey C Thomasson[4,5], Elizabeth B Farquhar[2,6], Jessica A McCoppin[4], Karl D Castillo[2,7]

[1] The Department of Biology, Boston University, Boston, Massachusetts, USA
[2] Environment, Ecology, and Energy Program, The University of North Carolina at Chapel Hill, Chapel Hill, North Carolina, USA
[3] Department of Marine and Environmental Sciences, Northeastern University, Nahant, MA, USA
[4] The Department of Biology, The University of North Carolina at Chapel Hill, Chapel Hill, North Carolina, USA
[5] Florida Fish and Wildlife Conservation Commission, St. Petersburg, Florida, USA
[6] Center for Marine Science, University of North Carolina Wilmington, Wilmington, NC, USA
[7] The Department of Marine Science, The University of North Carolina at Chapel Hill, Chapel Hill, North Carolina, USA
[*]*Corresponding author:* colleenbove@gmail.com

Table A. Number samples per species per treatment assessed in physiological and plasticity analyses. Note that plasticity sample sizes are smaller due to comparison within colony resulting in reduced samples when a control fragment was not present.

		Physiology N		Plasticity N	
	**Treatment**	**Offshore**	**Inshore**	**Offshore**	**Inshore**
Porites astreoides					
current day (uatm)	28C	6	6		
current day (uatm)	31C	2	4	2	4
end-of-century (uatm)	28C	6	6	5	6
end-of-century (uatm)	31C	0	4	0	4
extreme (uatm)	28C	5	5	5	5
extreme (uatm)	31C	3	5	3	5
pre industrial (uatm)	28C	6	5	5	5
pre industrial (uatm)	31C	3	3	2	3
Pseudodiploria strigosa					
current day (uatm)	28C	3	2		
current day (uatm)	31C	3	2	0	0
end-of-century (uatm)	28C	9	6	4	2
end-of-century (uatm)	31C	5	3	2	1
extreme (uatm)	28C	8	6	3	2
extreme (uatm)	31C	3	2	2	0
pre industrial (uatm)	28C	10	6	4	2
pre industrial (uatm)	31C	5	4	2	1
Siderastrea Siderea					
current day (uatm)	28C	6	5		
current day (uatm)	31C	5	6	5	5
end-of-century (uatm)	28C	6	6	6	5
end-of-century (uatm)	31C	6	6	6	5
extreme (uatm)	28C	7	5	7	4
extreme (uatm)	31C	6	5	6	4
pre industrial (uatm)	28C	6	4	6	4
pre industrial (uatm)	31C	3	4	3	4

Table B. PERMANOVA model assessment for best-fit model selection of PCAs per species. Akaike information criterion (AIC) was used to select the best-fit model per species. For all species, the fully additive model was the best-fit model (temperature + pCO_2 + reef).

Species	Full interactive model AIC	Best fit (additive) model AIC
S. siderea	692.3	678.5
P. strigosa	696.9	685.8
P. astreoides	500.5	497.1

Table C. Model performance comparisons of generalized linear mixed effects models (GLMM) for plasticity assessments to select the best-fit model per species using the package *performance* (version 0.8.0). Akaike information criterion (AIC) was used to select the best-fit model per species. The models highlighted in grey were used for bootstrapping estimates and 95% confidence intervals.

Model formula	AIC	Conditional R^2	Marginal R^2
Siderastrea Siderea			
reef environment * pCO2 * temperature + (1 | colony)	223.2	0.545	0.366
reef environment * pCO2 + temperature + (1 | colony)	218.8	0.506	0.322
reef environment * pCO2 + temperature + (1 | colony) + (1 | tank)	218.4	0.542	0.307
reef environment + pCO2 * temperature + (1 | colony)	225.6	0.442	0.253
reef environment + pCO2 + temperature + (1 | colony)	221.6	0.442	0.254
reef environment * (pCO2 + temperature) + (1 | colony)	220.1	0.511	0.329
pCO2 + temperature + (1 | colony)	222.1	0.37	0.088
Pseudodiploria strigosa			
reef environment * pCO2 * temperature + (1 | colony)	110.7	0.427	0.347
reef environment * pCO2 + temperature + (1 | colony)	106	0.341	0.292
reef environment + pCO2 * temperature + (1 | colony)	106.9	0.313	0.271
pCO2 + temperature + (1 | colony)	102.8	0.268	0.224
Porites astreoides			
reef environment * pCO2 * temperature + (1 | colony)	153.1	0.527	0.199
reef environment * pCO2 + temperature + (1 | colony)	145.9	0.521	0.195
reef environment + pCO2 * temperature + (1 | colony)	146.2	0.5	0.174
reef environment + pCO2 + temperature + (1 | colony)	142.3	0.499	0.174
reef environment * (pCO2 + temperature) + (1 | colony)	147.9	0.522	0.195
pCO2 + temperature + (1 | colony)	140.4	0.485	0.147
pCO2 + temperature + (1 | colony) + (1 | tank)	142.4	0.493	0.145

Table D PERMANOVA model output from each species using the *adonis2* function with 1500 iterations.

	Df	Sum of Squares	R2	F	P-value
Siderastrea Siderea					
pCO_2	3	59423	0.203	7.93	0.00067
temperature	1	9320	0.032	3.73	0.05463
reef environment	1	24705	0.084	9.89	0.00133
Residual	*80*	*199740*	*0.682*		
Total	*85*	*292988*	*1*		
Pseudodiploria strigosa					
reef environment	1	101796	0.09	14.87	0.00067
temperature	1	519372	0.46	75.84	0.00067
pCO_2	3	30444	0.027	1.48	0.22252
Residual	*71*	*486202*	*0.43*		
Total	*76*	*1130099*	*1*		
Porites astreoides					
reef environment	1	724	0.005	0.53	0.48568
temperature	1	27051	0.191	19.66	0.00133
pCO_2	3	30537	0.216	7.4	0.00067
Residual	*62*	*85309*	*0.603*		
Total	*67*	*141417*	*1*		

Table E. GLMM output from plasticity assessments for each species. The intercept of each model was set as 300 µatm, 28 °C, and inshore reef environment.

Species		Estimate	Standard error	Statistic	P-value
S. siderea	*(Intercept)*	1.085	0.172	6.3	0
	reef environment (offshore)	-0.058	0.248	-0.24	0.814
	pCO$_2$-current	0.335	0.173	1.94	0.053
	pCO$_2$-EOC	0.21	0.131	1.6	0.109
	pCO$_2$-extreme	0.419	0.132	3.17	0.002
	temperature (31°C)	0.003	0.076	0.04	0.967
	reef environment (offshore):pCO$_2$-current	-0.704	0.239	-2.95	0.003
	reef environment (offshore):pCO$_2$-EOC	-0.409	0.197	-2.08	0.037
	reef environment (offshore):pCO$_2$-extreme	-0.278	0.191	-1.45	0.146
	Conditional R^2	*0.506*			
	Marginal R^2	*0.322*			
P. strigosa	*(Intercept)*	1.279	0.148	8.66	0
	pCO$_2$-EOC	-0.338	0.193	-1.75	0.08
	pCO$_2$-extreme	-0.059	0.187	-0.31	0.753
	temperature (31°C)	0.227	0.173	1.31	0.19
	Conditional R^2	*0.232*			
	Marginal R^2	*0.188*			
P. astreoides	*(Intercept)*	1.038	0.121	8.61	0
	pCO$_2$-current	-0.047	0.11	-0.43	0.67
	pCO$_2$-EOC	0.032	0.075	0.44	0.664
	pCO$_2$-extreme	0.122	0.078	1.57	0.116
	temperature (31°C)	0.264	0.065	4.07	0
	Conditional R^2	*0.485*			
	Marginal R^2	*0.147*			

Table F. PERMANOVA model output across species using the *adonis2* function with 1500 iterations.

	Df	Sum of Squares	R^2	F	P-value
pCO_2	3	149393	0.04	8.24	0.0007
temperature	1	17313	0	2.87	0.0933
reef environment	1	58058	0.02	9.61	0.0047
species	2	1642613	0.42	135.9	0.0007
temperature:species	2	553351	0.14	45.78	0.0007
pCO_2:species	6	90865	0.02	2.51	0.024
reef environment:species	2	77259	0.02	6.39	0.004
Residual	*214*	*1293204*	*0.33*		
Total	*231*	*3882055*	*1*		

Table G. PERMANOVA model output of coral host or algal symbiont physiology per species using the *adonis2* function with 1500 iterations depicted in **Figures S8-S10**.

	**Coral host**					**Algal symbiont**				
	**Df**	**Sum of Squares**	**R^2**	**F**	**P-value**	**Df**	**Sum of Squares**	**R^2**	**F**	**P-value**
S. siderea										
pCO_2	3	1	0.019	0.55	0.74883	3	61056	0.208	8.15	0.00067
temperature	1	3	0.075	6.65	0.00333	1	7468	0.025	2.99	0.10127
reef environment	1	0	0.006	0.52	0.57295	1	24705	0.084	9.9	0.00266
Residual	*80*	*30*	*0.901*			*80*	*199684*	*0.682*		
Total	*85*	*34*	*1*			*85*	*292913*	*1*		
P. strigosa										
pCO_2	3	1	0.041	1.23	0.3058	3	26899	0.024	1.31	0.28181
temperature	1	3	0.147	13.12	0.00067	1	515173	0.456	75.24	0.00067
reef environment	1	0	0.02	1.75	0.15656	1	101793	0.09	14.87	0.00067
Residual	*71*	*14*	*0.793*			*71*	*486140*	*0.43*		
Total	*76*	*18*	*1*			*76*	*1130005*	*1*		
P. astreoides										
pCO_2	3	2	0.136	3.48	0.01532	3	29037	0.205	7.04	0.00133
temperature	1	0	0.036	2.76	0.08328	1	26338	0.186	19.15	0.00067
reef environment	1	0	0.021	1.64	0.18121	1	724	0.005	0.53	0.47768
Residual	*62*	*10*	*0.807*			*62*	*85288*	*0.603*		
Total	*67*	*13*	*1*			*67*	*141387*	*1*		
