# Supplementary material for: Global change differentially modulates Caribbean coral physiology
Source: PLoS One. 2022 Sep 2;17(9):e0273897. doi: 10.1371/journal.pone.0273897 (PMC9439252; doi:10.1371/journal.pone.0273897)
Supplement: Attachment — Submitted filename: Review of Bove et al._PLoS One.docx [file pone.0273897.s012.docx]

**Manuscript Number:** PONE-D-22-07335R1
**Manuscript Title:** Global change differentially modulates Caribbean coral physiology and suggests future ‘winners’ and ‘losers’

**General comments:**

This paper is a revised version of an initial submission, though this reviewer was not one of the two original reviewers. Overall, I think this paper is improved from its original version based on the previous reviewers’ comments and the tracked changes. I do think this paper deserves to be published in PLoS ONE, but recommend some additional minor revisions prior to acceptance.

I appreciate the Github link for the data and analysis codes used for this paper.

**Specific comments:**

I think the introduction is well-written, especially after seeing the additions made from the first round of review, but I have a few additional comments.

Line 119: I would classify S.sid as “weedy” with P.ast

Line 119: in reference to line 435 in the discussion – in the discussion the authors write “Indeed, *P. strigosa* is known to be a more thermally sensitive coral species…” but in line 119 in the intro you write that P.strig was chosen b/c it’s stress-tolerant. This needs to be clarified.

Line 119: Maybe the authors could consider expanding upon the framing of the 3 selected species – we know that coral cover has declined rapidly on Caribbean reefs due to a myriad of factors, most recent of which include SCTLD. Perhaps framing the experiment a little more intentionally, e.g., you selected weedy/stress tolerant spp because you wanted to test what the predominant responses of the likely-to-be-dominant corals on reefs of the Caribbean will be… it’s not far off from what you have now, but right now it feels like it’s implied – like you didn’t write that but I’m guessing that’s what you intended. If you’re more intentional with the rationale and the “why it matters”, it just makes the reader more invested in reading on.

Truthfully, when I first read this paper, my initial thought was this deserves to be published; however, I’m not sure how novel it is. Lots of labs have done cross-factorial TxCO_2_ work like this to assess physiological responses in corals and their symbionts between 2012-present (other Bove papers, other Castillo lab papers, Baumann, Grottoli, Schoepf, Towle, Okazaki, Manzello, Enochs, Edmunds, and many more…) But the framing of this is what might set this paper apart from the many that came before it.

We know the Caribbean is in trouble. We know we have lost a lot of major reef builders. The question that persists is: for the weedy and stress tolerant spp – is there hope for them under combined and possibly extreme TXCO_2_ scenarios, and on top of that – at prolonged stress exposure, e.g. 90 days, not just 30 or 60 days? I’d like to see the authors play that up and distinguish how this work is novel compared to the many previous studies that at first glance might seem very similar. Convince us why this experiment matters – why we still need this research as a contribution to the literature. The authors start to get at this in lines 593-599 in the Discussion, but I think expanding it could really strengthen the paper.

Methods:

Line 163, Table 1: Are there two typos?

In the preindustrial CO_2_ column should the **31** +/- 96 be 301?

Similarly in the current-day CO_2_ column should the **47** +/- 152 be 407?

Line 169-170: Is there a reason why the authors combined frozen artemia and newly hatched artemia? Why not just keep the feedings consistent by using one or the other? Doesn’t that technically introduce another variable? In reality, I’m sure there was no measurable effect on heterotrophy between frozen and freshly hatched, but you never know…? Also – how much artemia were fed to each tank? Quantity is important to mention here when thinking about how feeding affects lipids, etc., even though feeding rate was not tested in this study. In short, I noticed the sentence (line 169-170) was added following the first review, but I still think it could use an additional sentence or two of clarification about amount (quantity) of heterotrophic food offered to the corals in each treatment tank. Do the authors know approximate zooplankton density on the natal reefs in Belize? Or was the amount fed based on previous studies?

Results:

I noticed a couple of inconsistencies with italicizing and using the subscript “2” for CO_2_ so I recommend the authors just do a find and replace for species names and “CO2”. Examples include line 316 (species names not italicized) and line 320 “pCO2”. I also noticed use of the British spelling for “colour”. I think as long as it’s consistent it’s fine, and I’m sure if the journal wants the use of “color” that can be corrected during final formatting/editing, but just flagging that the authors might want to check.

Line 369: Do you have any ideas about why the *P. strigosa* controls had low survivorship? Do you attribute it to transfer stress form Belize to Massachusetts? Having low survivorship in the controls is a reason to potentially be more critical (or at least skeptical) of treatment results, so if the authors have any ideas about this, they should be briefly mentioned/discussed.

Line 401: Does “constrained physiology” mean “less plastic”?

Discussion:

Line 435: see comment above from introduction about the incongruence with the statement in line 119.

Line 494-510: I found this paragraph a little hard to get through. There’s so much use of the word “plasticity” and the positive and negative aspects of how plasticity manifested in P. ast and P. strig in the experiment. Then the authors distinguish between the concept of “physiological plasticity” from the study and genotypic plasticity (not analyzed in this study). I get what the authors are trying to convey, but the wording and flow of this paragraph (at least for me) could be improved to help the reader more quickly grasp the take-home messages.

Line 590-593: I generally agree with this statement. I realize you tested more than one parameter in this study, but you still proceeded to use the “winner” and “loser” terminology in your article title after cautioning others against this (for good reason)…

Lines 593-599: See earlier comment about expansion of these ideas.
